# Psychological capital, quality of life, and well-being in mother caregivers of individuals with down syndrome

**DOI:** 10.3389/fpsyg.2023.1145104

**Published:** 2023-02-21

**Authors:** Alina Chiracu, Germina-Alina Cosma, Amalia Raluca Stepan, Marian Alexandru Cosma, Ionuț Corlaci, Eleonora Daniela Ciupeanu Călugăru, Florin Voinea, Mihaela Zăvăleanu, Horia Alin Burileanu, Taina Avramescu

**Affiliations:** ^1^Faculty of Psychology and Educational Sciences, University of Bucharest, Bucharest, Romania; ^2^Faculty of Physical Education and Sport, University of Craiova, Craiova, Romania; ^3^Faculty of Physical Education and Sport, National University of Physical Education and Sports, Bucharest, Romania; ^4^Faculty of Physical Education and Sport, Ovidius University of Constanța, Constanța, Romania

**Keywords:** psychological capital, quality of life, well-being, down syndrome, caregivers

## Abstract

**Introduction:**

Caused by an error in cell division that produces an additional chromosome 21, Down syndrome (DS) is one of the most common developmental disorders in the world. This study aims to analyze the relationship between psychological capital, quality of life and well-being of caregivers of individuals with Down syndrome (DS).

**Methods:**

The participants were 98 caregivers (mothers, *M* = 52.13, *SD* = 11.39) of individuals with Down syndrome. The instruments used were the Psychological Capital Questionnaire (measuring self-efficacy, resilience, optimism, and hope), Quality of Life Questionnaire (including social support, general satisfaction, physical/psychological health, absence of excessive workload/free time), and Psychological Wellbeing Scale, investigating the following dimensions: self-acceptance, positive relationships with others, autonomy, environmental mastery, purpose in life, and personal growth.

**Results:**

The mediation analysis showed that self-efficacy, hope, and resilience are positively associated to quality of life, and optimism is positively associated to well-being. The total effects of psychological capital on well-being are positive and significant and quality of life mediates the relationship between psychological capital and well-being.

**Discussion:**

These results show that psychological capital is an important inner resource for caregivers of DS individuals and must be improved through support services, so that caregivers have a higher perception of the quality of life and implicitly of well-being.

## 1. Introduction

Developmental disabilities are long-term psychological and physiological impairments which affect an individual’s ability to perform activities of daily living, such as independent feeding, communicating, and mobilizing (World Health Organization and Unicef, 2012). Down syndrome (DS) is one of the most common developmental disorders in the world. In the European Union, out of 5.2 million births per year, approximately 104,000 (2.5%) have congenital anomalies. DS has a share of 8% of all congenital anomalies (European Commission Report, 2019). There are no relevant statistics in Romania on the number of people with DS, but given that worldwide one in 700 children is born with DS (de Graaf et al., 2015), it is estimated that there are currently over 4,000 people with DS living in Romania. A preliminary statistic made through a project financed by the European Commission within Youth in Action program shows that there are currently 4,420 people with DS in Romania, of which 2,611 children and 1,809 adults (Apopei Pruteanu, 2022).

DS is a disorder caused by an error in cell division that produces an additional chromosome 21, present in every cell of the body. Because of this additional genetic material, the disorder is also called Trisomy 21 (Ghosh et al., 2009). DS is often accompanied by impairments in cognitive ability and physical growth, neurodevelopmental and behavioral disorders, mood and anxiety disorders (Moyal et al., 2014; Jonsson et al., 2017), developmental disabilities, a higher risk for a series of health problems, including heart diseases, problems with the digestive system or limbs, thyroid dysfunction, hearing and visual disturbances, and obstructive sleep apnea (Bull, 2011; Alexander et al., 2016). These determine the inability of people with DS to take care of themselves or to have a normal and autonomous functioning in the activities of daily living, which requires the presence of a permanent caregiver (Henn et al., 2008; Areias et al., 2011; El-Gilany et al., 2017).

People with DS have a lower rate of development and require greater parental involvement, which influences family dynamics (Most et al., 2006; Corrice and Glidden, 2009). Although this affects the whole family, the primary caregiver is the one who assumes full responsibility for providing physical, emotional, and drug assistance. Most studies point out that this role is most often played by the mother (Greenberg et al., 2004; Stoneman, 2007). Raising and caring for a child or an adult with disabilities, including DS, is an additional effort for the caregiver and the family in general because individuals with DS continue living with their parents also into adulthood (McKenzie et al., 2016). For this reason, the quality of life and well-being of caregivers should be a priority in social programs and policies (Masefield et al., 2020). It is widely acknowledged that family caregivers are more vulnerable to psychological distress, with higher rates of depression and anxiety (Liu et al., 2018; Connors et al., 2019; Teahan et al., 2021).

On the other hand, caring for a loved one can be a rewarding experience. The ability to build a positive perspective on the situation and the related tasks can lead to a decrease in the perception of burden and thus to an improvement in the quality of life, with positive effects on well-being (Bertrand, 2019). Caregiver’s positive attitude toward his/her life context is based on a number of personal characteristics, which allow him to integrate reality as a challenge. It is well known that people with DS need special, permanent care that extends throughout their entire lives and becomes more and more complicated as they get older (Carvalho et al., 2015; McCarron et al., 2018). Therefore, the caregiver must possess or develop a set of attributes and skills that will support him in coping with the high demands of caring for a person with a disability. Psychological capital may be an important strength of caregivers of people with DS.

### 1.1. Psychological capital

Psychological capital (PsyCap) represents a set of individual characteristics and qualities that express a person’s positive resources (Luthans and Avolio, 2003). This construct contributes to the successful fulfillment of the responsibilities of daily life, through confidence in one’s abilities, proactive actions, optimistic approach to life and the future, and persistence in overcoming obstacles (Santisi et al., 2020). PsyCap has four dimensions: self-efficacy, hope, resilience, and optimism (Luthans et al., 2007), thus being generated by the individuals’ belief that they are able to strive to achieve their goals, to persevere in achieving these goals, and mobilize efforts and activities in this regard, resilience to difficult situations and flexibility in recovery of balance after experiencing them, and positive attitude regarding achievement of success at present or in the future (Luthans, 2002; Luthans and Youssef-Morgan, 2017). Self-efficacy is defined as one’s confidence in his or her ability to mobilize the motivation, cognitive resources, and courses of action necessary to achieve certain levels of performance (Stajkovic and Luthans, 1998). Individuals with high self-efficacy will generalize their existing expectations of their abilities to perform tasks in different undertakings (Bandura, 1997) and in new situations (Sherer et al., 1982), being more useful when facing unknown challenging situations (Grether et al., 2018). Resilience is the capacity to bounce back from adversity, uncertainty, failure, and adapt to changing and stressful life demands (Masten and Reed, 2002; Tugade and Fredrickson, 2004). High resilience has been associated with good physical and mental health (Schure et al., 2013). Optimism refers to an individual’s expectancy of positive outcomes (Scheier et al., 2001). Many studies have shown the beneficial aspects of optimism for different domains of life, such as physical health, prevention of depression, effective decision-making, or life satisfaction (Aspinwall, 2005; Magnano et al., 2015; Santilli et al., 2017). Hope is based on the interaction between two factors: goal-directed energy and pathways (Snyder et al., 1996). Higher hope is associated with specific goal-setting and goal-accomplishment behaviors that may increase the likelihood of successful goal attainment (Cheavens et al., 2019). All these positive psychological attributes can be improved and play an important role in individuals’ personal growth.

### 1.2. Quality of life

Quality of life (QoL) is a concept used in many contexts, being correlated with health and well-being. After 1980, the content of the concept expanded to include the subjective experience of the individual regarding social life, daily activity, and health (Moreno and Ximenez, 1996), being defined as the subjective perception of individuals of their position in life (Church, 2004; Eiser et al., 2004). WHO defines QoL as an individual’s perception of their position in life, in the context of the value systems and the culture in which they live, and in relation to their expectations, goals, concerns, and standards (World Health Organisation Quality of Life Assessment Group. (WHOQOL), 1994, 1998). This broad-ranging concept incorporates the person’s psychological state, physical health, independence level, personal beliefs and values, social relationships, and, also, relationship to salient characteristics of the environment [World Health Organisation Quality of Life Assessment Group. (WHOQOL), 1994]. It follows from this definition that QoL does not refer to the real, objective conditions of a person’s life, but to the personal experience of those conditions, representing the degree of satisfaction with regard to family, love, and social life, as well as the environment (Minayo et al., 2000). Life satisfaction, happiness, and experienced well-being are mutually interrelated, being closely linked to perceived quality of life (Păunescu et al., 2018).

### 1.3. Well-being

Well-being is a construct that derives from positive psychology and refers to cultivating positive emotions to ensure the optimal functioning of individuals (Campbell et al., 1976; Ryan and Deci, 2001; Culbertson et al., 2010). Well-being refers to the development of skills and personal growth, its origins being found in concepts such as self-actualization (Maslow, 1968) or full functionality (Rogers, 1961). Ryff C. D. (1989) proposed a multidimensional model of well-being that includes six dimensions: self-acceptance, positive relationships with others, autonomy, environmental mastery, purpose in life, and personal growth. By self-acceptance, people try to feel good about themselves, while good relationships with others and the ability to love are characteristics of positive mental activity and mental health (Keyes et al., 2002). Autonomy refers to maintaining one’s own beliefs, authority, and personal independence in different contexts of life to strengthen one’s identity (Ryff and Keyes, 1995). Environmental mastery is the ability of the individual to create and choose environments that meet their personal needs and desires, and purpose in life is the need for people to set goals and pursue their goals (Keyes et al., 2002). The well-being of patients and their caregivers and close family members has been shown to be closely intertwined (Martire et al., 2004), suggesting that supporting the caregiver’s well-being will positively affect the health of the patient, and vice versa.

### 1.4. Relationships among psychological capital, quality of life, and well-being

The developmental character of the components of PsyCap is supported by the fact that their presence determines “who you are here and now,” but also “who you can become” in the future (Jurek and Niewiadomska, 2021).

Self-efficacy is the belief in the ability to use one’s own cognitive resources and skills and to make the necessary efforts to successfully complete a task (Bandura, 1997). People with high levels of self-efficacy interpret the hardships of life as challenges, have greater decision-making ability, are more motivated to be involved in proactive behaviors, and have control over their actions, which leads to success, to satisfaction with the tasks performed and to an adaptive and constructive coping (Bandura et al., 1999; Bandura, 2008; Diseth, 2011; Larson et al., 2013). All these lead to the idea that these individuals will have higher levels of quality of life and implicitly higher well-being.

Hope reflects the person’s belief that he will succeed and that his success depends on his ability to plan and use alternative ways to achieve success and to overcome and avoid those ways that do not lead to success (Snyder, 2002). This leads to a sense of being in control of events and the ability to assess difficult situations as less threatening, determining an increase in motivation to initiate remedial actions (Avey et al., 2009). Hope plays an important role in setting goals and striving to achieve them. This mechanism leads to efficient behaviors, to a positive adaptation and implicitly to an increase in the likelihood of experiencing high quality of life and high well-being (Schulz et al., 2014; Rabenu and Yaniv, 2017).

Resilience contributes to a flexible adaptation to the demands of life, in an appropriate, consistent, and persistent way, both by adjusting one’s abilities and by judiciously using environmental factors (Bonanno, 2004). Positive adaptation is reflected in the fact that the person is able to cope in a positive way with adversities, failures, traumatic events, and also with growing responsibility. Thus, resilience reflects the person’s ability to endure adverse events and then return to their initial state of balance and functionality after experiencing these adverse events. People with high levels of resilience are characterized by self-confidence, independence, sense of humor, patience, positive emotions, openness to new experiences, and determination in action, which contributes to increased quality of life and well-being (Dorfman and Rubenstein, 1994; Luoh and Herzog, 2002; Dingemans et al., 2016).

Optimism is defined as the tendency to perceive, explain, and evaluate life in a positive rather than negative terms and to predict future events as fortunate rather than unfortunate (Peterson and Seligman, 1984). Optimistic people tend to interpret negative events as external, temporary, and situational, and positive events as personal, long-lasting, and universal. Optimism is a personal disposition, being accompanied by positive emotions, leading to greater involvement in goal-oriented activities, supporting the motivation to succeed, and thus improving the quality of life and well-being of the individual (Carver and Scheier, 2002; Carver et al., 2005).

PsyCap plays an important role in the personal development and growth of individuals (Newman et al., 2014). It facilitates the attention and memory processes needed to successfully overcome obstacles and achieve well-being (Diener and Biswas-Diener, 2008) and enhances the ability of individuals to manage adverse situations, which increases quality of life and well-being (Sweetman and Luthans, 2010). Positive relationships between PsyCap and quality of life have been found by many researchers (Luthans et al., 2007; Hansen et al., 2015).

A theoretical approach to the characteristics of caregivers of people with DS may be the Adaptation Hypothesis, which assumes that repeated exposure to the challenges of caring for people with various disorders increases and not decreases personal resources (Townsend et al., 1989). Two processes can be involved here: successful fulfillment of adjacent responsibilities can lead to increased skills, self-confidence and self-esteem, and exposure to stress can provide opportunities for personal well-being (Seltzer et al., 2004). One of the best-known theories that explains how individuals cope with the demands of caregiving was developed by McCubbin and Patterson (1983). The model has been extensively used in research on the care of people with disabilities, explaining the positive results by the presence of personal resources at stake, but also by the significance that people attribute to the events they face and the actions they take. Resources can include personal skills and attributes, such as PsyCap, but, also, financial and social capital. Therefore, PsyCap is closely related to QoL, and, also, to well-being.

QoL and well-being are often used interchangeably and inconsistently within studies (De Leo et al., 1998), primarily because of their objective and subjective components. Langlois and Anderson (2002) argue that QoL results from the congruence between the resources provided by the environment and the needs expressed by individuals, and well-being refers to the dynamic processes that lead to better conditions in life. QoL involves variables such as aspiration and recollection and is more neutral, while well-being is the positive physical, social, and mental state that stems from a series of collective goods and relationships with people and places (Mohit, 2014). Well-being presupposes that basic psychological needs are met and are determined by conditions such as supportive personal relationships, community empowerment, good health, financial security, rewarding employment, and healthy and attractive environment (Rosly and Abdul Rashid, 2013).

### 1.5. The present study

With a strong PsyCap, the caregivers can change their perspective on their own life, so as to report high levels of QoL, which will lead to a high level of well-being. Through its components, self-efficacy, hope, resilience, and optimism, PsyCap becomes an inner resource of caregivers, contributing to increasing the quality of their lives and the positive perception of the present and the future.

Taking into account the above, the following hypothesis was established:

*H1*: Quality of life mediates the relationship between psychological capital and well-being among caregivers of individuals with DS.

## 2. Methods

### 2.1. Participants and procedure

Participants were 98 caregivers (mothers) of individuals with DS, all of them women, aged between 25 and 76 years (*M* = 52.13*, SD* = 11.39). The age of individuals with DS ranged between 4 and 42 years (*M =* 23.53*, SD =* 10.83) and the duration of caregiving ranged between 1 and 41 years (*M =* 18.80*, SD =* 11.13). Socio-demographic profiles of the participants are presented in Table 1 (the study took place in Romania).

**Table 1:** Socio-demographic profile of caregivers.

Variable	Total n	Total %
Education level		
Secondary or less	11	11
Higher education	87	89
Monthly income		
< 2,500 RON	43	44
2,500–5,000 RON	46	56
> 5,001 RON	9	9
Marital status		
Single	19	19
Married/in a relationship	79	81
Residence area		
Rural	22	22
Urban	76	78
Occupation		
Caregiver only	45	46
Caregiver and other	53	54

After receiving the institutional approval (from the Research Ethics Committee of the University of Craiova, protocol no. 1/08.02.2021), recruitment partners were developed consisting in organizations and schools who had relationships with DS individuals’ families. These included disability and social service providers, advocacy and support providers, and educational service providers. Participants were recruited online and invited to participate in the study. We provided email invitations, asking partners to reach parents for the recruitment announcement. Of 150 invited caregivers, only 98 agreed to participate in the study (65% response rate). Data were collected between June and December 2021. The questionnaires were administered online. The first section of the form contained a brief description of the study, the informed consent of the participants, and the GDPR agreement. The study was conducted in accordance with the Declaration of Helsinki. In the current research, the snowball sampling technique was used to investigate mother caregivers of individuals with DS, a hard-to-reach population, difficult to involve in public health programs or research (see Shaghaghi et al., 2011).

### 2.2. Instruments

*Socio-demographics*—data were collected on nine variables related to demographics of caregivers: gender, age, educational level (1—secondary education or less, 2—higher education), monthly income (1— < 2,500 RON, 2–2,500–5,000 RON, 3— > 5,001 RON, 1 USD = 4.69 RON on 3 January 2023), marital status (1—single, 2—married or in a relationship), residence area (1—rural, 2—urban), occupation (1—caregiver only, 2—caregiver and other occupation), caregiving duration, as well as data on age of individuals with DS.

*Well-being* was measured with Psychological Well-Being Scale (Ryff C. 1989), Romanian version (Kállay and Rus, 2014). The measure consists of 42 items and measures six dimensions of well-being: self-acceptance (e.g., “When I look at the story of my life, I am pleased with how things have turned out”), positive relationships with others (e.g., “Maintaining close relationships has been difficult and frustrating for me”), autonomy (e.g., “My decisions are not usually influenced by what everyone else is doing”), environmental mastery (e.g., “The demands of everyday life often get me down”), purpose in life (e.g., “I enjoy making plans for the future and working to make them a reality”), and personal growth (e.g., “I think it is important to have new experiences that challenge how you think about yourself and the world”). Responses are provided on a six-step Likert scale, where 1—*strongly disagree* and 6—*strongly agree* (there are reverse-scored items, for example, if the scored is 2, the adjusted score is 5, 1 becomes 6 and so on.). In the present study, we used the global score. The global score was calculated by summing the responses to all the 42 items; it ranges between 42 and 252, with an average value of 147; high scores reflect an increased level of well-being.

*Psychological capital* was measured with Psychological Capital Questionnaire (PCQ; Luthans et al., 2007). The questionnaire consists of 24 items, six for each of the four dimensions: self-efficacy, hope, resilience, and optimism. Responses are provided on a seven-step Likert scale, where 1—*total disagreement* and 7—*total agreement*. The scores for each dimension were calculated by arithmetic means on the answers of corresponding items; scores ranged between 1 and 7, with higher values reflecting higher levels of self-efficacy, hope, resilience, and optimism. Items example: self-efficacy—“I feel confident analyzing a long-term problem to find a solution;” hope—“I can think of many ways to reach my current (work) goals;” resilience—“I usually manage difficulties one way or another (at work);” optimism—“When things are uncertain for me (at work) I usually expect the best.” For the purpose of this study, those words that refer strictly to work and the organizational environment were removed from the items of the questionnaire, thus adapting the items for the activities of daily living.

*Quality of life* was measured with Quality of Life Questionnaire (QoLQ; Boixadós et al., 2009). Responses are provided on a five-step Likert scale, where 1—*never* and 5—*always*. The survey consists of 39 items and measures the quality of life, having four dimensions: social support (e.g., “Do you feel that you have someone to turn to when you need company or support?”), general satisfaction (e.g., “Do you feel that life is meeting your expectations?”), physical/psychological health (e.g., “Do you feel that you are in good health?”), and absence of excessive workload/free time (e.g., “Does your work leave you enough free time for other things that you want to do?”). In the present study, we used the global score. The global score was calculated by summing the responses to all the 39 items; it ranges between 39 and 195, with an average value of 117; high scores reflect an increased level of quality of life.

In the case of PCQ and QoLQ, the questionnaires were translated into Romanian by the authors of the current study in accordance with the rules of translation-retroversion-adaptation, a method that has been used in other studies (e.g., Makarowski et al., 2021; Piotrowski et al., 2021). The reliability coefficients (McDonald’s omega - ω) of the scales (considering the three questionnaires), in the present research, can be seen in Table 2.

**Table 2:** Means, standard deviations, Cronbach Alpha coefficients, and correlations among variables.

	M	SD	α	SE	HO	RE	OP	QoL	WB
SE	5.22	1.67	0.90	1					
HO	5.89	0.86	0.83	0.62^**^	1				
RE	5.58	0.99	0.78	0.43^**^	0.70^**^	1			
OP	5.44	0.90	0.73	0.58^**^	0.74^**^	0.72^**^	1		
QoL	148.24	25.12	0.95	0.76^**^	0.74^**^	0.61^**^	0.68^**^	1	
WB	184.40	28.88	0.92	0.65^**^	0.74^**^	0.65^**^	0.79^**^	0.79^**^	1

^**^p < 0.01; SE – Self-efficacy, HO – Hope, RE – resilience, OP – Optimism, QoL – Quality of life, WB – Well-being.

### 2.3. Data analyses

All data were exported from Google sheet into SPSS.24 (IBM Corp, Released 2016, IBM SPSS Statistics for Windows, Version 24.0. Armonk, NY). Descriptive statistics and Pearson’s correlation were executed through SPSS, while mediation analysis and the reliability of the scales were executed through Jamovi (The jamovi project, 2021). The effect size index for correlation, *r*^2^ (the coefficient of determination) is interpreted as follows: *r*^2^ = 0.09—a moderate effect; *r*^2^ = 0.01—a small effect; and *r*^2^ = 0.25—a large effect (Cronk, 2020).

## 3. Results

Means, standard deviations, McDonald’s omega coefficients for the scales of the current study, and correlations among variables are presented in Table 2.

Skewness and kurtosis range between −1.12 and 1.36, denoting a normal distribution of data (George and Mallery, 2016).

Overall, caregivers reported high levels of psychological capital, with the highest score for hope (*M* = 5.89*, SD* = 0.86), followed by resilience (*M =* 5.58*, SD = 0*.99), by optimism (*M =* 5.44, *SD = 0*.90), and finally by self-efficacy (*M =* 5.22*, SD =* 1.67). At the same time, the ratings for quality of life (*M =* 148.24*, SD =* 25.12) and for well-being (*M =* 184.40*, SD =* 28.88) are more than average.

The correlations among variables are strong. The caregivers with high self-efficacy report also high quality of life (*r* = 0.76, *p* < 0.01, *r*^2^ = 0.58) and high well-being (*r* = 0.65, *p* < 0.01, *r*^2^ = 0.42); the caregivers with high level of hope report high quality of life (*r* = 0.74, *p* < 0.01, *r*^2^ = 0.55) and high well-being (*r* = 0.74, *p* < 0.01, *r*^2^ = 0.55); the caregivers with high resilience report high quality of life (*r* = 0.61, *p* < 0.01, *r*^2^ = 0.37) and high well-being (*r* = 0.65, *p* < 0.01, *r*^2^ = 0.42); and the caregivers with high optimism report high quality of life (*r* = 0.68, *p* < 0.01, *r*^2^ = 0.46) and high well-being (*r* = 0.79, *p* < 0.01, *r*^2^ = 0.62).

### 3.1. Hypothesis testing

To test the research hypothesis, a mediation analysis was performed, with the four dimensions of psychological capital (self-efficacy, hope, resilience, and optimism) as predictors, well-being as a dependent variable and quality of life as a mediator. The indirect, direct and total effects of psychological capital on well-being and the component relationships among variables are presented in Table 3.

**Table 3:** Mediation analysis for quality of life on the relationship between psychological capital dimensions and well-being.

Type	Effect	Estimate	SE	95% C.I.		*β*	z	*p*
				Lower	Upper			
Indirect	PCSE ⇒ QoL ⇒ WB	3.30	0.90	1.53	5.08	0.20	3.65	0.00
	PCHO ⇒ QoL ⇒ WB	3.48	1.40	0.75	6.22	0.11	2.50	0.01
	PCRE ⇒ QoL ⇒ WB	1.98	0.98	0.05	3.91	0.07	2.01	0.04
	PCOP ⇒ QoL ⇒ WB	1.57	1.14	−0.66	3.81	0.05	1.38	0.17
Component	PCSE ⇒ QoL	6.71	1.10	4.55	8.87	0.46	6.09	0.00
	QoL ⇒ WB	0.49	0.11	0.28	0.70	0.43	4.57	0.00
	PCHO ⇒ QoL	7.07	2.37	2.42	11.72	0.25	2.98	0.00
	PCRE ⇒ QoL	4.01	1.79	0.50	7.53	0.16	2.24	0.03
	PCOP ⇒ QoL	3.19	2.21	−1.13	7.52	0.12	1.45	0.15
Direct	PCSE ⇒ WB	0.31	1.38	−2.39	3.02	0.02	0.23	0.82
	PCHO ⇒ WB	3.21	2.65	−1.98	8.40	0.10	1.21	0.23
	PCRE ⇒ WB	3.05	1.96	−0.80	6.90	0.11	1.55	0.12
	PCOP ⇒ WB	10.11	2.38	5.45	14.78	0.33	4.25	0.00
Total	PCSE ⇒ WB	3.62	1.30	1.06	6.17	0.21	2.78	0.01
	PCHO ⇒ WB	6.69	2.81	1.19	12.19	0.21	2.38	0.01
	PCRE ⇒ WB	5.03	2.12	0.87	9.18	0.18	2.37	0.02
	PCOP ⇒ WB	11.69	2.61	6.58	16.80	0.38	4.48	0.00

SE – Self-efficacy, HO – Hope, RE – resilience, OP – Optimism, QoL – Quality of life, WB – Well-being.

The indirect effect of self-efficacy on well-being is positive and significant, *b* = 3.30, CI95%(1.53, 5.08), *β* = 0.20, z = 3.65, *p* < 0.01; the indirect effect of hope on well-being is positive and significant, *b* = 3.48, CI95%(0.75, 6.22), *β* = 0.11, *z* = 2.50, *p* < 0.05; the indirect effect of resilience on well-being is positive and significant, *b* = 1.98, CI95%(0.05, 3.91), *β* = 0.07, *z* = 2.01, *p* < 0.05; and the indirect effect of optimism on well-being is insignificant, *b* = 1.57, CI95%(−0.66, 3.81), *β* = 0.05, *z* = 1.38, *p* = 0.17.

The component relationships among variables show that quality of life is positively associated with well-being, *b* = 0.49, CI95%(0.28, 0.70), *β* = 0.43, *z* = 4.57, *p* < 0.01, self-efficacy is positively associated with quality of life, *b* = 6.71, CI95%(4.55, 8.87), *β* = 0.46, *z* = 6.09, *p* < 0.01, hope is positively related to quality of life, *b* = 7.07, CI95%(2.42, 11.72), *β* = 0.25, *z* = 2.98, *p* < 0.01, resilience is positively associated with quality of life, *b* = 4.01, CI95%(0.50, 7.53), *β* = 0.16, *z* = 2.24, *p* < 0.05, but optimism is not linked to quality of life, *b* = 3.19, CI95%(−1.13, 7.52), *β* = 0.12, *z* = 1.45, *p* = 0.15.

The direct effects of psychological capital on well-being are insignificant for self-efficacy, *b* = 0.31, CI95%(−2.39, 3.02), *β* = 0.02, *z* = 0.23, *p* = 0.82, for hope, *b* = 3.21, CI95%(−1.98, 8.40), *β* = 0.10, *z* = 1.21, *p* = 0.23, for resilience, *b* = 3.05, CI95%(−0.80, 6.90), *β* = 0.11, *z* = 1.55, *p* = 0.12, and positive and significant for optimism, *b* = 10.11, CI95%(5.45, 14.78), *β* = 0.33, *z* = 4.25, *p* < 0.01.

When talking about the total effects of psychological capital on well-being, these are positive and significant. For self-efficacy, *b* = 3.62, CI95%(1.06, 6.17), *β* = 0.21, *z* = 2.78, and *p* < 0.05; for hope, *b* = 6.69, CI 95% (1.19, 12.19), *β* = 0.21, *z* = 2.38, and *p* < 0.05; for resilience, *b* = 5.03, CI 95% (0.87, 9.18), *β* = 0.18, *z* = 2.37, and *p* < 0.05; and for optimism, *b* = 11.69, CI 95% (6.58, 16.80), *β* = 0.38, *z* = 4.48, and *p* < 0.01.

## 4. Discussions

There is no doubt that caregivers of people with Down syndrome (DS) are prone to high levels of stress, given that DS is a lifelong disorder and that caregiver responsibilities increase as the person with DS gets older. However, families and caregivers are gradually developing certain personality attributes and skills that become essential resources in adapting to their life context. In line with the purpose of the current research, the relationship between psychological capital, quality of life, and well-being of caregivers of individuals with DS were investigated. The descriptive analysis showed that the participants reported high levels for all dimensions of PsyCap and well-being, as well as for QoL. Statistical analysis showed that three out of the four dimensions of PsyCap are positively associated with QoL (self-efficacy, hope, and resilience, but not optimism) and only one dimension (optimism) is associated with well-being—the direct effect, when talking about the mediation analysis. QoL is a significant mediator in the relationships between self-efficacy, hope, resilience, and well-being, but not in the relationship between optimism and well-being. The total effects are, anyway, positive and significant, QoL being a significant mediator in the relationship between PsyCap and well-being in caregivers/mothers of individuals with SD.

These results can be attributed to the fact that caregivers are people who successfully integrate the responsibilities they have in relation to the individual with DS. The challenges posed by this situation lead to the development of traits and skills that lead to personal growth and good management of stressful or critical situations. High levels of self-efficacy, hope, and resilience can make caregivers feel that they have a normal life, perceiving a more than average QoL and benefiting from all its components: meaningful work, acceptable family relationships, time for self-care, and good health. This positive perception of QoL leads to a higher well-being, the caregivers being satisfied with their level of autonomy, their control over the environment, the quality of interpersonal relationships, have high levels of self-acceptance, personal growth, and maybe the most important, considering meaning of life. It should be noted that the participants are mothers of individuals with DS. In a family with a member with DS daily routines change, the home adjusts, relationships and priorities change, but in essence, life takes its course, and the responsibilities generated by this situation become real challenges that must be successfully overcome. A number of studies showed that mothers of children with DS experienced better well-being and less burden than mothers of children with other intellectual disabilities (Hodapp et al., 2001; Hodapp, 2002). The above-average results for Qol and well-being could be attributed to marital status, 81% of mothers being married and having a stable relationship. Previous studies reported that married couples split the care and responsibilities, which can contribute to a higher well-being (Zajicek-Farber et al., 2015).

Optimism was not significantly associated with QoL, which may be due to the fact that QoL is a variable that refers to the concrete living conditions of the individual, to the way he/she lives the daily life, and optimism can diminish the pragmatic nuance of caregivers’ existence, who have increased responsibilities in the family. Conversely, optimism is positively associated with well-being, which reflects its subjective nature. Well-being implies the subjective perception of the reality in which the individual lives his life, thus it is natural that optimism contributes to a higher well-being, allowing the caregiver to view life in a positive light. Optimism has a special contribution in building well-being because it predisposes the person to a positive approach to the life situations. None of the other three dimensions of PsyCap were directly associated with well-being, but only through the mediation exerted by QoL. Manzano-Garcia and Ayala (2017) obtained similar results in a study on the relationship between PsyCap and well-being among specialists working with people with autism. As mentioned authors asserted, PsyCap is a valuable personal resource and through its components helps the caregiver to successfully cope with the demands, obstacles, and adversities they face. In the same direction, Larue (2014), in a study on social workers working with traumatized people, analyzed the relationship between PsyCap and well-being, as well as the mediating role of flexible coping. Their results showed that only PsyCap is responsible for the high level of well-being, but not flexible coping. On the other hand, Isa et al. (2016) showed that adaptive coping contributes to increasing the well-being of caregivers of children with disabilities (especially optimism, acceptance, and religiosity). Self-efficacy, as a component of PsyCap, has also been shown to be an important resource in maintaining a high level of well-being and even of health state (Rezendes and Scarpa, 2011; Guillamón et al., 2013). Also, Truitt et al. (2012) showed that hope (another component of PsyCap) contributes significantly to increasing adaptation to uncertainty among caregivers of children with DS, which implicitly leads to increased well-being.

We consider that psychological capital can provide a viable solution to enhance the quality of life and well-being of caregivers of individuals with DS. The power of people to move forward and face life’s obstacles is immeasurable. If at the level of individuals with Down syndrome, there are implemented programs aimed to improve some components of the quality of live (Cosma et al., 2017; Barbu et al., 2021), counseling activities of caregivers should be taken into consideration with more involvement, at least in Romania. Support groups can be set up for caregivers to talk about their own experiences, about the obstacles they have faced and about the ways in which they have overcome them. This sharing of information and the search for the normalization of their life experiences will facilitate a better understanding and adaptation to the role of caregiver and will probably increase overall psychological capital. In addition, these meetings can help increase socialization and thus improve the quality of life with positive implications for well-being. Support groups may be moderated by psychologists or other specialists to further inform caregivers and suggest ways to overcome mental barriers.

The present research has some limitations. One of the limitations refers to the exclusive participation of women and the online application of the questionnaires (explicit measures/self-report tools assume the issue of desirable answers, aspect known in the literature; see Predoiu et al., 2022). However, the relatively large number of mother caregivers of individuals with DS tested represents a strength of the research. Another limitation is the *ex post facto* design, a longitudinal study, in the future, being more relevant in order to follow the evolution, over time, of psychological capital, perceived quality of life, and caregivers’ well-being, differentiating, also, between those with a shorter duration and those with a longer duration of caregiving activity. Also, further studies could examine, separately, dimensions of well-being, such as self-acceptance, autonomy, positive relationships with others, environmental mastery, purpose in life, personal growth, and dimensions of quality of life—general satisfaction, social support, physical/psychological health, and absence of excessive workload/free time. Not least, the results might be different if only younger (or older) women were investigated, or taking into account the age of individuals with DS (only children, teenagers, or adults).

## 5. Conclusion

In summary, caregivers (mothers of individuals with DS) reported high levels of psychological capital, with the highest score for hope, followed by resilience, optimism, and self-efficacy. People with higher levels of PsyCap are better able to handle adversities and challenging situations, so they can perceive a higher QoL and have a higher level of subjective well-being. Considering the perceived quality of life and well-being the results are, also, above average. An indirect effect of self-efficacy, hope, and resilience on caregivers’ well-being was highlighted. Quality of life was positively related to well-being, hope, self-efficacy and resilience, but not with the optimism level, while the direct effects of psychological capital on well-being are insignificant for self-efficacy, hope, resilience, but significant for optimism. PsyCap dimensions (SE, HO, and RE) were directly associated with well-being, but only through the mediation exerted by QoL.

## Data availability statement

The raw data supporting the conclusions of this article will be made available by the authors, without undue reservation.

## Ethics statement

The studies involving human participants were reviewed and approved by Research Ethics Committee of the University of Craiova, protocol no. 1/08.02.2021. The patients/participants provided their written informed consent to participate in this study.

## Author contributions

All authors listed have made a substantial, direct, and intellectual contribution to the work and approved it for publication.

## Conflict of interest

The authors declare that the research was conducted in the absence of any commercial or financial relationships that could be construed as a potential conflict of interest.

## Publisher’s note

All claims expressed in this article are solely those of the authors and do not necessarily represent those of their affiliated organizations, or those of the publisher, the editors and the reviewers. Any product that may be evaluated in this article, or claim that may be made by its manufacturer, is not guaranteed or endorsed by the publisher.
